# The perspective of modern transplant science – transplant arteriosclerosis: inspiration derived from mitochondria associated endoplasmic reticulum membrane dysfunction in arterial diseases

**DOI:** 10.1097/JS9.0000000000002362

**Published:** 2025-03-28

**Authors:** Jingyi Li, Qian Lin, Chao Ren, Xiaodong Li, Xiaowei Li, Haofeng Li, Shadan Li

**Affiliations:** a Department of Urology, The General Hospital of Western Theater Command, Chengdu, Sichuan, China; b Department of General Surgery (Vascular Surgery), The Affiliated Hospital of Southwest Medical University, Luzhou, Sichuan, China; c College of Medicine, Southwest Jiaotong University, Chengdu, Sichuan, China

**Keywords:** chronic transplant dysfunction, endothelial cells, mitochondria-associated endoplasmic reticulum membrane, transplant arteriosclerosis, vascular smooth muscle cells

## Abstract

The mitochondria-associated endoplasmic reticulum membrane (MAM) is a crucial structure connecting mitochondria and the endoplasmic reticulum (ER), regulating intracellular calcium homeostasis, lipid metabolism, and various signaling pathways essential for arterial health. Recent studies highlight MAM’s significant role in modulating vascular endothelial cells (EC) and vascular smooth muscle cells (VSMC), establishing it as a key regulator of arterial health and a contributor to vascular disease pathogenesis.

Organ transplantation is the preferred treatment for end-stage organ failure, but transplant arteriosclerosis (TA) can lead to chronic transplant dysfunction, significantly impacting patient survival. TA, like other vascular diseases, features endothelial dysfunction and abnormal proliferation and migration of VSMC. Previous research on TA has focused on immune factors; the pathological and physiological changes in grafts following immune system attacks have garnered insufficient attention. For example, the potential roles of MAM in TA have not been thoroughly investigated.

Investigating the relationship between MAM and TA, as well as the mechanisms behind TA progression, is essential. This review aims to outline the fundamental structure and the primary functions of MAM, summarize its key molecular regulators of vascular health, and explore future prospects for MAM in the context of TA research, providing insights for both basic research and clinical management of TA.

**HIGHLIGHTS**

- Mitochondria-associated endoplasmic reticulum membrane (MAM), as a connecting structure between mitochondria and the endoplasmic reticulum, plays a significant regulatory role in arterial diseases.
- MAM participates in the occurrence and progression of arterial diseases through lipid metabolism, calcium homeostasis, mitochondrial dynamics, and glucose metabolism.
- The mechanism by which MAM regulates arterial diseases provides a foundation for research on transplant arteriosclerosis.

## Introduction

Organ transplantation is widely recognized as the primary therapeutic approach for patients with end-stage organ failure ^[^1-3^]^. Despite the advances in transplantation techniques and immunosuppressive therapies, chronic transplant dysfunction (CTD) continues to pose a significant challenge to the long-term survival of transplant recipients^[^4^]^. Transplant arteriosclerosis (TA) is the core pathological feature of CTD that is the leading cause of allograft failure^[^5^]^. The histological characteristics of TA include endothelial dysfunction, inflammation around the graft vasculature, loss of vascular smooth muscle cells (VSMC) in the media, formation of neointima, and deposition of the extracellular matrix^[^6-9^]^. Studies have shown that the occurrence of TA is closely related to the damage and subsequent repair responses experienced by graft vessels following immune attacks. Specifically, the immune migration of host-derived T cells and macrophages to the adventitia leads to scar formation and loss of vascular compliance. This condition subsequently triggers the migration and proliferation of VSMC into the intima. This dysfunction results in neointimal hyperplasia and lumen narrowing, ultimately reducing graft blood flow and leading to interstitial fibrosis and allograft failure^[^10-12^]^. Despite extensive research in this field, which involves signaling pathways such as TGF-β/Smad3, PI3K/Akt, AMPK/mTOR, and MAPK/ERK – all of which are related to endothelial apoptosis and abnormal VSMC behavior^[^13-16^]^ – further investigation into the mechanisms involved in TA is still needed. Recent perspectives suggest that although TA originates from immune attacks on the vascular system of the graft, its progression is also associated with non-immune factors present in the subsequent “injury-repair” processes. These factors, such as mitochondrial dysfunction, ischemia-reperfusion injury and abnormal repair mechanisms, may further exacerbate the pathological state of TA^[^17^]^. Recent perspectives suggest that TA primarily arises from immune damage to the vascular system, but non-immune factors contributing to endothelial injury and inflammation may also exacerbate this damage, further worsening the pathological state of TA^[^17^]^.

Studies have shown that arterial diseases are closely associated with the disruption of mitochondrial and the endoplasmic reticulum (ER) homeostasis^[^18,19^]^. Mitochondria not only play a crucial role in energy production, but also regulate intercellular communication and participate in autophagy and apoptosis^[^20^]^. Homeostatic disruption of the ER, an important cellular signaling hub and protein synthesis factory, can significantly impact cellular functions and contribute to the progression of atherosclerotic diseases^[^21-25^]^. Despite the distinct roles of mitochondria and the ER in cellular function, their interaction is mediated by the mitochondria-associated endoplasmic reticulum membrane (MAM). This concept was first proposed in 1969 and defined as an independent intracellular structure in 1990^[^26,27^]^. Dysfunction of MAM can lead to aging^[^28^]^, and regulate various diseases in the cardiovascular system^[^29,30^]^, nervous system^[^31-33^]^, urinary system^[^34-36^]^, musculoskeletal system^[^37,38^]^, reproductive system^[^39,40^]^, and plays a crucial role in vascular health and the progression of arterial diseases^[^41^]^. This review will focus on the structure and function of MAM in arterial diseases.

## Structure and function of MAM

**Figure 1.**
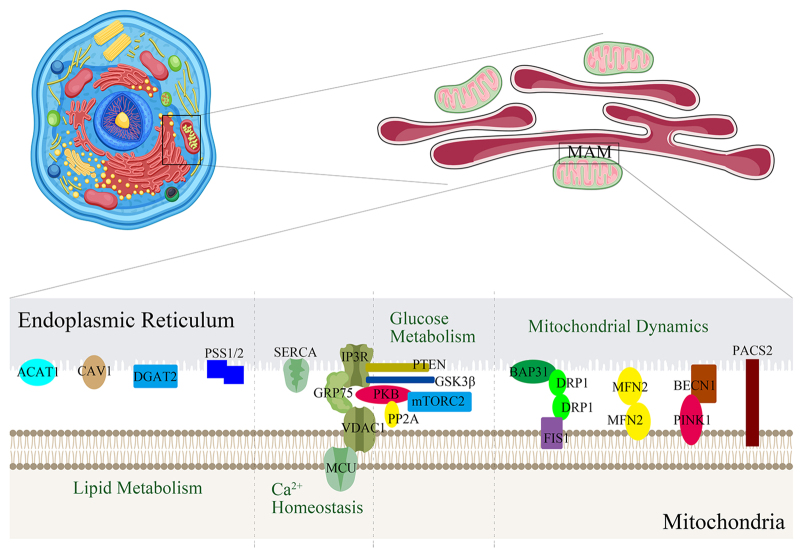
Regulatory proteins for arterial diseases in MAM.

The distance between the ER and mitochondria is approximately 10-25 nm, and they interact through membrane proteins at their contact sites while maintaining independent structures^[^42^]^. With advances in research techniques, such as real-time imaging, electron microscopy, and subcellular fractionation, researchers have been able to observe and confirm the existence of the contact region between the ER and mitochondria, referred to as MAM^[^43^]^. Electron tomography has revealed that MAM is composed of numerous proteins that are situated between the ER and the outer mitochondrial membrane^[^44^]^. These proteins, functioning analogously to “ropes” and “bridges,” regulate the contact distance between the two organelles and mediate the exchange of lipids, calcium ions, and other signaling molecules, thereby influencing cellular functions^[^45^]^. Therefore, the proper functioning of MAM depends on maintaining an appropriate distance between the mitochondria and the ER, and if this distance is too small or too large, the functionality of MAM will be disrupted^[^46^]^. Researchers have extracted thousands of proteins from MAM, most of which are highly conserved rather than having truly specific functions^[^47,48^]^. Further in-depth investigations have revealed that only about 68 proteins are potentially functionally active within MAM^[^49^]^. These proteins can be broadly categorized based on their functions: lipid metabolism, Ca^2+^ homeostasis, mitochondrial dynamics, glucose metabolism, as well as ER stress, autophagy, apoptosis, and inflammation^[^45^]^. Wang *et al* proposed that aging is associated with abnormal autophagy, while cancer and cardiovascular diseases are linked to calcium homeostasis imbalance^[^28^]^. Xu *et al* suggested that neurodegenerative diseases are associated with lipid metabolism abnormalities, dysregulation of calcium homeostasis, and ER stress^[^32^]^. Elwakiel *et al* suggested that type 2 diabetes and diabetic nephropathy are closely related to abnormalities in glucose metabolism, lipid metabolism, and ER stress^[^34^]^. Zheng *et al* and Grepper *et al* observed calcium homeostasis imbalance in lesions of the locomotor system^[^37,38^]^, while Guo *et al* and Wang *et al* found similar findings in primary reproductive system damage^[^39,40^]^. Li *et al* showed that mitochondrial dynamics-related genes play a functional role in renal ischemia-reperfusion injury^[^35^]^. Ren *et al* found evidence of ER stress in heart failure^[^29^]^, while Congur *et al* reported similar findings in diabetic cardiomyopathy^[^30^]^. Additionally, Zhang *et al* also revealed evidence of ER stress in depression^[^33^]^, and Ke *et al* identified this stress in urolithiasis^[^36^]^. Yu *et al* revealed that neuronal cells in neurodegenerative diseases, including Alzheimer’s disease, display abnormalities in autophagy^[^31^]^. These latest research findings indicated that these functional abnormalities of MAM are implicated in the pathogenesis of various diseases (Table 1). This article will focus on reviewing the first four functions that are primarily related to arterial diseases (Fig. 1).

**Table 1:** Latest findings in the diseases linked to MAM

Dysfunction	Diseases
Lipid metabolism	Type 2 diabetes
	Diabetic nephropathy
	Neurodegenerative changes
Ca^2+^ homeostasis	Cardiovascular disease
	Intervertebral disc degeneration
	Skeletal muscle dysfunction
	Neurodegenerative changes
	Cancer
	Primary reproductive system injury
Mitochondrial dynamics	Renal ischemia-reperfusion injury
Glucose metabolism	Type 2 diabetes
	Diabetic nephropathy
ER stress	Urolithiasis
	Heart failure
	Neurodegenerative changes
	Depression
	Type 2 diabetes
	Diabetic cardiomyopathy
	Diabetic nephropathy
Autophagy	Aging
	Alzheimer’s disease

### Key physiological functions of MAM associated with arterial lesions

**Figure 2.**
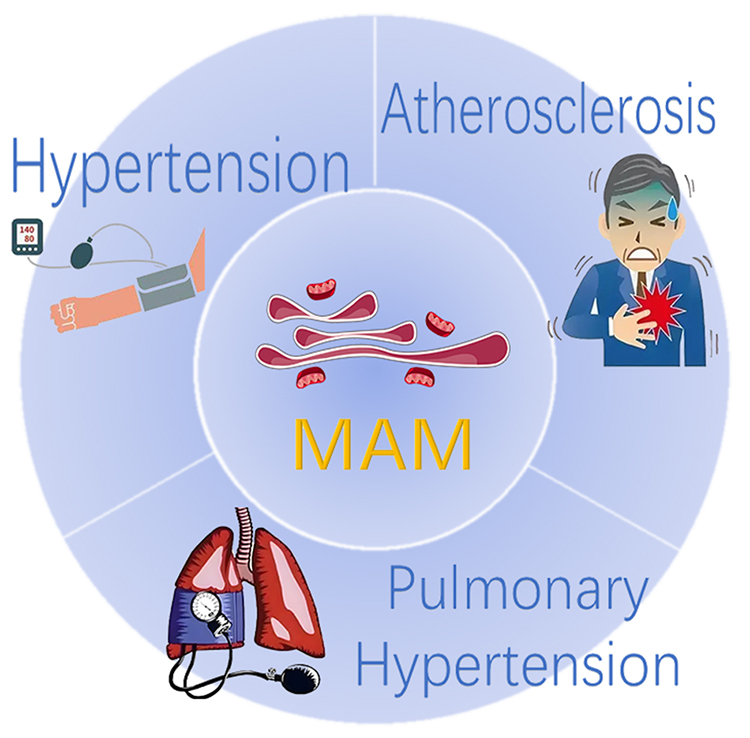
MAM-related arterial diseases.

**Figure 3.**
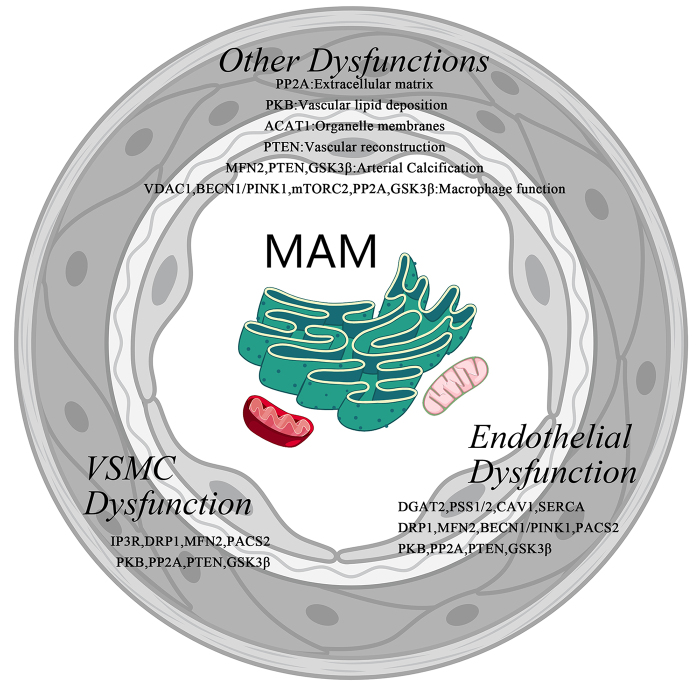
MAM proteins and arterial lesions.

MAM plays a critical role in maintaining cellular homeostasis and regulating biological functions, many of which are closely associated with the onset and progression of arterial diseases (Table 2, Figs. 2 and 3).

**Table 2:** Regulatory proteins for arterial diseases in MAM

Function types	Proteins	Abbreviation	Relevant functions in arterial diseases
Lipid metabolism	Acyl-coenzyme A:cholesterol acyltransferase 1	ACAT1	Regulate cholesterol accumulation in organelle membranes
	Diacylglycerol O-acyltransferase 2	DGAT2	Regulate EC function
	Phosphatidylserine synthases 1 and 2	PSS1/PSS2	Regulate the vascular endothelial barrier
	Caveolin-1	CAV1	Regulate EC function
Ca^2+^ homeostasis	Sarcoplasmic/endoplasmic reticulum Ca^2+^ ATPase	SERCA	Regulate EC function
	Inositol 1,4,5-trisphosphate receptor	IP3R	Regulate VSMC function
	Voltage dependent anion channel 1	VDAC1	Regulate macrophage function
Mitochondrial dynamics	Dynamin-related protein 1	DRP1	Regulate EC/VSMC function
	Mitofusin 2	MFN2	Regulate EC/VSMC function
			Induce arterial calcification
	Beclin 1 & PTEN induced kinase 1	BECN1 & PINK1	Regulate macrophage/EC function
	Phosphofurin acidic cluster sorting protein 2	PACS2	Regulate EC/VSMC function
Glucose metabolism	Protein kinase B	PKB	Regulate the vascular endothelial barrier & VSMC function & vascular lipid deposition
	Mammalian target of rapamycin complex 2	mTORC2	Regulate macrophage function
	Protein phosphatase 2A	PP2A	Regulate EC/PDGF/VSMC /macrophage function & extracellular matrix
	Phosphatase and tensin homolog	PTEN	Regulate EC/VSMC function & the vascular endothelial barrier
			Induce arterial calcification
			Vascular reconstruction
	Glycogen synthase kinase-3β	GSK3β	Regulate EC/VSMC/macrophage function
			Induce arterial calcification

### Lipid metabolism

Several proteins involved in lipid metabolism are present on MAM, playing important roles in the biosynthesis and transport of phospholipids^[^50^]^.

ACAT1 is a protein that resides in the ER and accumulates on MAM, where it converts cholesterol into cholesterylesters. Inhibiting ACAT1 function can lead to the accumulation of cholesterol in organelle membranes, potentially affecting cellular function and delaying the progression of atherosclerosis (AS)^[^51,52^]^.

DGAT2 is an enzyme localized to the ER that catalyzes the synthesis of triglycerides (TG) in eukaryotes. When TG synthesis increases, DGAT2 accumulates on MAM^[^53^]^. Inhibiting the DGAT2 pathway may improve AS by preventing endothelial damage^[^54^]^.

On MAM, PSS1/PSS2 is responsible for the synthesis and transport of phosphatidylserine (PS), yet with minimal expression in the ER^[^55,56^]^. Research has shown that a treatment targeting PS can promote the reconstruction of the vascular endothelial barrier, offering therapeutic benefits for AS^[^57^]^.

CAV1, a resident protein of MAM, regulates intracellular cholesterol transport^[^58^]^. Its overexpression may induce dysfunction in pulmonary artery endothelial cells (EC), and targeting this pathway may help reduce pathological vascular remodeling^[^59^]^.

Proteins such as PEMT2 and ACS4 are localized to MAM and are responsible for regulating lipid metabolism^[^60,61^]^, however, there are currently no reports linking these proteins to arterial health, and therefore, we will not elaborate on this matter here.

### Calcium homeostasis

The ER and mitochondria play central roles in calcium ion signaling, with MAM serving as an important structure for calcium ion transfer^[^62^]^.

The calcium pump SERCA, primarily localized to the ER, is enriched in the MAM, where it is responsible for transporting calcium ions from the cytosol into the ER, thereby maintaining intracellular calcium homeostasis^[^63^]^. Inhibiting SERCA induces ER stress in EC, while adenosine monophosphate (AMP)-activated protein kinase (AMPK), a physiological inhibitor of ER stress, alleviates the progression of AS by maintaining SERCA activity and intracellular calcium ion homeostasis^[^64^]^.

Within MAM, the IP3R-GRP75-VDAC1-MCU complex exists as a functional unit, facilitating the transfer of calcium ions from the ER to the mitochondria^[^65^]^. Upon activation, IP3R on the ER membrane releases calcium ions from the ER lumen, These ions are then taken up by VDAC1 on the outer mitochondrial membrane, with the assistance of GRP75. Finally, calcium ions enter the mitochondrial matrix through the mitochondrial calcium uniporter (MCU), a channel located on the inner mitochondrial membrane^[^66^]^. The calcium ion signaling fluxes generated in this process has significant biological implications. Specifically, IP3R, a transmembrane protein on the ER membrane, is primarily responsible for the release of calcium ions^[^67^]^. GRP75 (Glucose-regulated protein 75), located on the outer mitochondrial membrane, facilitates the interaction between IP3R and VDAC1, promoting calcium ions exchange between the ER and mitochondria^[^65,68^]^. VDAC1, an outer mitochondrial membrane protein, is an important component of the mitochondrial permeability transition pore (mPTP) and is essential for regulating mitochondrial function in vascular cells^[^69,70^]^. During the progression of AS, apolipoprotein E (APOE) may interact with VDAC1, leading to mitochondrial dysfunction^[^71^]^. Targeting VDAC1 has been proposed as a strategy to regulate macrophage apoptosis, which may have implications for AS treatment^[^72^]^. MCU is a transmembrane protein located on the inner mitochondrial membrane that mediates calcium ion transport within mitochondria^[^73^]^. Overall, the hyperactivation of this functional complex can lead to an abnormal accumulation of calcium ions within the mitochondria, resulting in the opening of the mPTP and subsequent apoptosis. Notably, IP3R can regulate VSMC proliferation and migration, processes closely related to arterial diseases^[^74^]^.

Additionally, other proteins, such as SIG-1R and CYPD, regulate calcium ion channels^[^75,76^]^. However, there remains a lack of research regarding their associations with arterial diseases.

### Mitochondrial dynamics

The connection between the ER and mitochondria is an essential mechanism for regulating cellular functions, with several key proteins involved in this process.

DRP1 is primarily located in the cytoplasm, with a small amount present on the ER. It maintains the connection between the ER and mitochondria^[^77^]^, and is also involved in the formation of atherosclerotic plaques in AS^[^78^]^. In MAM, DRP1 functions as part of the BAP31-FIS1-DRP1 complex. The B cell receptor-associated protein 31 (BAP31), located on the ER membrane, and mitochondrial fission 1 protein (FIS1), found on the outer mitochondrial membrane, form this complex. When mitochondrial fission begins, DRP1 binds to the BAP31-FIS1 complex at MAM, further inducing mitochondrial fragmentation and degradation^[^79,80^]^. Inhibition of DRP1 can lead to vascular endothelial damage due to increased mitochondrial reactive oxygen species (ROS)^[^81^]^, while the proliferation of VSMC decreases due to reduced mitochondrial fission^[^82^]^. These EC and VSMC abnormalities may play a central role in the development of vascular remodeling and arterial diseases, thereby making DRP1 a potential target for treatment.

MFN2, a protein located on the outer mitochondrial membrane, directly regulates the contact between the ER and mitochondria. When MFN2 is deficient, mitochondrial calcium ion uptake is impaired, in contrast, its overexpression can induce apoptosis^[^83^]^. MFN2 also facilitates the transport of PS to the mitochondria by binding to PS and promoting mitochondrial phosphatidylethanolamine (PE) synthesis, consequently, MFN2 deficiency can lead to reduced phospholipid synthesis, triggering ER stress^[^84^]^. Additionally, MFN2 can inhibit VSMC proliferation and reduce neointimal hyperplasia in graft veins^[^85^]^. Targeting MFN2 expression may promote neointimal formation by stimulating VSMC proliferation and migration^[^86-88^]^. However, it may also induce VSMC calcification, exacerbating vascular sclerosis^[^89,90^]^. Studies on the relationship between MFN2 expression and AS progression have yielded inconsistent results: some researchers have found that MFN2 expression levels positively correlate with AS progression^[^91^]^, while others suggest that overexpressing MFN2 can activate mitophagy, reduce EC apoptosis, and slow AS progression^[^92,93^]^. This indicates that, although MFN2 plays a significant role in arterial diseases, its regulatory mechanisms require further research and exploration.

The BECN1-PINK1 complex plays a crucial role in starvation-induced autophagy. Autophagosomes form at the MAM, where BECN1 and PINK1, located on the ER and mitochondria respectively, enhance the connectivity between these organelles. This increased connection further promotes autophagy and helps protect cellular functions^[^94,95^]^. PINK1 not only induces mitochondrial fission but also promotes VSMC proliferation, thereby exacerbating the progression of AS^[^96^]^. Conversely, the knockout of PINK1 reduces foam cell formation in macrophages, decreases EC apoptosis and oxidative stress, and helps counteract the progression of AS^[^97,98^]^.

PACS2 is a multifunctional sorting protein that regulates the association between the ER and mitochondria. Its deficiency disrupts MAM, induces ER stress, and elevates the levels of MAM-associated ACS4 and PSS1, thereby promoting apoptosis^[^99^]^. Studies have shown that knocking down PACS2 intensifies apoptosis in VSMC while inhibiting apoptosis in EC^[^41,100,101^]^.

The health of vascular EC and VSMC is influenced by the state of contact between the mitochondria and ER, with proteins such as DRP1, MFN2, PINK1, and PACS2 promoting VSMC proliferation. Inhibiting excessive contact between the ER and mitochondria also helps protect vascular EC^[^102^]^, suggesting that regulatory proteins involved in ER-mitochondria contact may serve as potential therapeutic targets for arterial diseases.

In addition, proteins such as INF2 and FUNDC1 have been identified as regulators of mitochondrial dynamics^[^103-105^]^. However, there is currently a lack of reports on their regulatory roles in vascular health.

### Glucose metabolism

MAM may represent a novel hub for insulin signaling^[^106^]^.

Recent findings indicate that PKB is also localized to MAM. Under insulin stimulation, PKB at the MAM interface is recruited and phosphorylated, resulting in the downregulation of IP3R channels, ultimately modulating MAM function^[^106^]^. In the context of vascular diseases, PKB promotes VSMC proliferation through multiple mechanisms^[^107-111^]^, impairs the function of the vascular endothelial barrier^[^112-114^]^, and contributes to increased lipid accumulation in the vasculature^[^115-118^]^, thereby affecting arterial health.

mTORC2 is a highly conserved protein kinase that localizes to MAM under growth factor stimulation. It regulates the phosphorylation of PKB at MAM. Phosphorylated PKB can influence PACS2 and IP3R proteins, thereby impacting various functions such as autophagy^[^119,120^]^. Moreover, mTORC2 also modulates macrophage activity, reduces macrophage adhesion to EC, and inhibits the progression of AS^[^121-123^]^. The role of mTORC2 in graft vasculature is receiving increasing attention^[^124^]^.

Impaired PP2A activity in MAM leads to increased phosphorylation and activation of PKB, downregulating IP3R channel activity and thereby affecting the overall function of the MAM^[^125^]^. Studies have shown that PP2A can alleviate EC dysfunction^[^126^]^, regulate EC inflammation and extracellular matrix components^[^127,128^]^, affect foam cell formation in transplanted macrophages^[^129-131^]^, and modulate platelet-derived growth factor (PDGF)^[^132^]^ as well as apolipoprotein E (APOE)^[^133^]^. Additionally, PP2A promotes neointimal fibrosis formation and inhibits abnormal VSMC proliferation^[^134-136^]^, helping to control the progression of arterial disease.

PTEN, a classic tumor suppressor gene, is partially localized to MAM and interacts with PKB and IP3R. PTEN antagonizes the effect of PKB on reducing calcium release through IP3R, thereby influencing apoptosis^[^137^]^. Furthermore, PTEN can impact EC function^[^92,138,139^]^, regulate VSMC proliferation and migration^[^140-142^]^, promote VSMC calcification, increase vascular endothelial inflammation, and modulate pathological vascular remodeling^[^143-145^]^. Through these actions, PTEN regulates the progression of arterial disease.

GSK3β is also localized to MAM and enhances IP3R function. Inhibiting GSK3β can reduce calcium ion flux, decrease cell sensitivity to apoptosis, and protect vascular EC^[^146-149^]^. GSK3β is also involved in regulating the expression of EC adhesion molecule, vascular calcification induced by endothelial-mesenchymal transition^[^150,151^]^, macrophage inflammatory cytokine production and infiltration^[^152,153^]^, and VSMC proliferation^[^154^]^. Additionally, it inhibits cholesterol efflux from macrophages, exacerbating AS^[^155^]^. GSK3β can also modulate the spatial polarity of EC, and its phosphorylation state significantly impact angiogenesis and revascularization^[^156,157^]^. Notably, studies suggest that nitric oxide (NO) facilitates the differentiation of perivascular progenitor cell (PPC) into brown adipocytes (BAT) via the activation of the solubilized guanylate cyclase/ protein kinase GIα (cGMP protein-dependent kinase Iα)/GSK3β pathways. This process may improve endothelial dysfunction in AS^[^158^]^.

Overall, factors involved in glucose metabolism influence the progression of arterial disease by affecting the functions of EC, VSMC, and macrophages. However, the role of MAM in this process still requires further research.

### Outlook

Common arterial diseases include Atherosclerosis, Arteritis, Aneurysms, Thrombotic Disorders, and Arterial Stenosis. Most of these conditions are closely associated with transplantation. The predominant pathological changes associated with hyperacute rejection of transplanted arteries include acute arterial thrombosis and necrotizing vasculitis. The main pathological alterations observed in acute rejection of transplanted arteries include arteritis and fibrinoid necrosis of the vascular wall. Chronic rejection of transplanted arteries primarily manifests as diffuse stenosis of the vascular lumen^[^9^]^. In clinical practice, the most prevalent and well-established practices for solid organ transplantation currently include kidney transplantation, liver transplantation, cardiac transplantation, and lung transplantation. However, during the postoperative management of kidney transplantation, transplant-related arterial sclerosis is significantly correlated with peritubular capillary leukocyte infiltration, glomerular inflammation, subclinical antibody-mediated rejection, and interstitial inflammation, which severely impact transplant kidney function and increase the incidence of adverse cardiovascular events in patients^[^11,159^]^. Ischemic cholangiopathy (IC) represents a significant cause of unfavorable outcomes after liver transplantation, with potential etiological factors including microvascular and macrovascular alterations in the transplanted liver, which are also classified as TA^[^160^]^ Cardiac allograft vasculopathy (CAV) represents a significant complication that adversely impacts patient survival following heart transplantation. It is classified as a form of TA, characterized by the gradual luminal narrowing of the coronary arteries post-transplantation, with its severity and progression closely correlated with lipid levels^[^161^]^. Following lung transplantation, chronic lung allograft dysfunction (CLAD) associated with TA similarly imposes significant limitations on the survival of recipients^[^162^]^. Under contemporary medical conditions, standardized management protocols are available for various types of rejection: comprehensive donor-recipient tissue matching can effectively prevent hyperacute rejection, while appropriate immunosuppressive therapy can reduce the incidence of T-cell-mediated acute rejection. However, the current strategies remain inadequate for addressing antibody-mediated chronic rejection (chronic transplant dysfunction)^[^163^]^.

**Figure 4.**
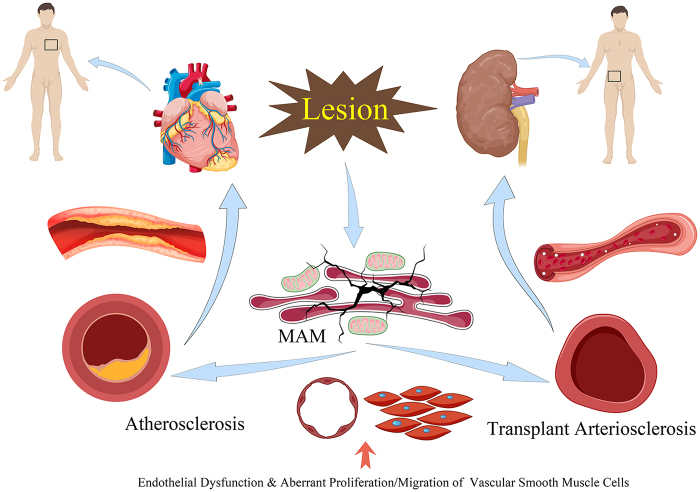
Common pathological changes between AS and TA.

Previous studies have illustrated that TA, as a defining pathological alteration of CTD, is initiated by antibody-mediated immune mechanisms^[^164^]^. However, as previously mentioned, the efficacy of current treatments, including immunosuppressive agents, plasmapheresis/immune adsorption therapy, intravenous immunoglobulin therapy, and glucocorticoids, remains suboptimal. Even the most advanced immunosuppressive agents fail to ensure the long-term functionality of transplanted organs. Furthermore, transplant arteries may experience the development of severe diffuse intimal hyperplasia within a timeframe of months to years, potentially obstructing graft blood flow and resulting in ischemia^[^17^]^. We have substantial grounds to hypothesize that there are factors, beyond the immune response, that interfere with the occurrence and progression of TA, and that these factors may play a crucial role. Regrettably, in the past two decades, scientific research on the pathological changes associated with TA has primarily concentrated on immune and immunological factors. Consequently, it is imperative to explore insights from alternative perspectives. TA is fundamentally an arterial disease characterized by dysfunction in endothelial cells and vascular smooth muscle cells^[^9^]^, this finding aligns with the pathophysiological changes documented in extensively researched vascular conditions, such as AS and post-angioplasty restenosis^[^165^]^, Hence, research focusing on the latter may offer valuable perspectives for the investigation of TA (Fig. 4).

It is noteworthy that there exists a significant amount of research on MAM in the context of AS. As previously stated, MAM affects the functions of endothelial cells and smooth muscle cells in the arteries through various mechanisms, including lipid metabolism, calcium homeostasis, mitochondrial dynamics, and glucose metabolism, thereby influencing the progression of AS. Given that the core pathological changes in AS and TA are nearly identical, we have reason to hypothesize that MAM may similarly regulate the occurrence and progression of TA. For instance, following transplant surgery, the arterial graft is exposed to factors such as surgical stress or immune attack, which trigger abnormalities mediated by MAM in lipid metabolism, calcium homeostasis, mitochondrial dynamics, and glucose metabolism in endothelial and smooth muscle cells. These abnormalities result in altered cellular behaviors, including proliferation and migration, which contribute to the onset and progression of TA. The molecular mechanisms and signaling pathways involved in this process merit further investigation. Pathophysiological alterations in subcellular organelles, including mitochondria and the endoplasmic reticulum, offer new research opportunities and therapeutic targets for the intervention and treatment of TA. This area represents a primary focus of both our current and future research endeavors.

In summary, MAM play a pivotal role in maintaining calcium homeostasis, regulating lipid and glucose metabolism, and monitoring the morphology and function of endoplasmic reticulum-mitochondria interactions. Their involvement is significant in the pathogenesis and progression of various arterial diseases. In the context of AS research, the role of MAM dysfunction in modulating arterial pathological changes has been extensively examined. However, current studies on TA reveal a relative paucity of investigation into vascular complications arising from dysfunction of subcellular organelles, including MAM. Consequently, we aim to elucidate the structure and function of MAM and provide a comprehensive overview of the key proteins that influence vascular endothelial cells, vascular smooth muscle cells, and other factors that may compromise vascular homeostasis. Through this endeavor, we hope to stimulate further exploration of the role of MAM in the progression of TA in future research and advocate for its consideration as a significant therapeutic target. This paper has certain limitations, primarily reflected in the fact that the proposed hypothesis currently lacks direct experimental evidence to support it, particularly there is a lack of evidence from TA patients or animal models that directly links MAM to TA. Given this situation, further direct research on TA is still needed to obtain more robust evidence to better support our hypothesis.

## Data Availability

The data used to support the findings of this study are included within the paper.
